# Chronic, self‐reported fatigue in adult immunotherapy patients and healthy controls: No association with cardiopulmonary exercise testing parameters

**DOI:** 10.14814/phy2.71090

**Published:** 2026-09-02

**Authors:** Damir Zubac, Timo Sonntag, Diana Kranjc, Freerk T. Baumann

**Affiliations:** ^1^ Department 1 of Internal Medicine, Center for Integrated Oncology Aachen, Bonn, Cologne, Düsseldorf University Hospital of Cologne Cologne Germany; ^2^ Science and Research Center Koper Institute for Kinesiology Research Koper Slovenia

**Keywords:** cardiotoxicity, immune system regulation, metabolic cost of cycling

## Abstract

This study examined whether cardiopulmonary exercise test (CPET) performance parameters are associated with chronic cancer‐related fatigue in patients receiving immunotherapy checkpoint inhibitors (ICI) and healthy controls. Also, the differences in energy expenditure of steady‐state cycling and gross efficiency were assessed during submaximal cycling efforts. This study involved patients (*n* = 19, 48 ± 13 years) receiving PD‐1, PD‐L1 checkpoint inhibitors and controls of similar age (*n* = 21, 49 ± 17 years). During the first visit, data on medical history, previous physical activity records, and CPET to voluntary exhaustion were collected. The second visit involved body composition assessment, the MFI‐20 fatigue questionnaire, and 30‐min steady‐state cycling at 90% gas exchange threshold. There were no significant differences observed between the groups in age, stature, BMI, or resting blood pressure. Peak V̇O_2_ was 33% lower, and the group under ICI treatment had lower cycling efficiency (22.49% vs. 20.07%, *p* = 0.031), compared to controls. All self‐reported fatigue items derived from MFI‐20 were significantly higher in the ICI group compared to controls (*p* = 0.001). The regression analysis found no association between the self‐reported general fatigue and data collected during CPET. Patients under ICI treatment reported higher levels of chronic fatigue, lower peak V̇O_2_, and reduced cycling efficiency. Interestingly, variables collected during CPET did not explain self‐reported chronic fatigue, underlying the complexity of this phenomenon.

## 1 INTRODUCTION

Over the last decade, the immune‐check point inhibitor (ICI) therapy has improved survival rates in several cancer diagnoses (Arafat Hossain, 2024); however, significant challenges regarding inflammatory toxicities and treatment side‐effects management still remain. According to a narrative review by Dougan et al. (2021), the most common inflammatory toxicities of the ICI treatment primarily affect endocrine organs, followed by the cardiac and neurological systems. In comparison to other systemic treatments (chemo‐ and radiotherapy), the immunotherapy treatment is more targeted, relying on the immune system's ability to distinguish between the cancer and normal cells. Activating the immune system in such way may trigger a variety of health issues, and these side effects are generally distinct from those of other systemic treatments (Dougan et al., 2021). For example, the ICI‐related toxicities can manifest at any time during the course of treatment (Milne et al., 2020). From the perspective of supportive care and the overall quality of life (QoL), ICI therapy is often associated with chronic fatigue, affecting ~30% of patients undergoing acute therapy (Lacey et al., 2019). Indeed, the contemporary clinical data suggest that patients undergoing ICI treatment, chronic fatigue is frequently linked to immune system dysregulation, persistent inflammation, and the onset of immune‐related adverse events. In contrast, chemotherapy‐induced fatigue is commonly linked to anemia, persistent inflammation, oxidative stress, and mitochondrial dysfunction. Apparently, the ICI‐related chronic fatigue tends to be more unpredictable compared to traditional anti‐cancer therapies, and some authors (Gomes et al., 2026; Hiensch et al., 2021; Wagner et al., 2025) suggest that exercise is a viable tool for mitigating chronic fatigue development, improving QoL and functional capacity. Still, there are no specific exercise protocols tailored for immunotherapy patients in the literature. The effects of structured exercise on chronic fatigue development in patients undergoing acute chemotherapy or radiotherapy have been extensively documented in the literature (Brownstein et al., 2022; Lacey et al., 2019; Mallard et al., 2025), and programs to increase exercise adherence are being developed (Huynh et al., 2026). The role of exercise in chronic fatigue management during ICI treatment remains reliant predominantly on self‐reported data (Lacey et al., 2019). What we currently know regarding exercise and immunotherapy treatment, is that exercise interventions are feasible and safe (Crosby et al., 2023), patients are often characterized with low physical activity levels (Pichel et al., 2025), poor aerobic capacity (Zubac et al., 2025), and data available in the literature are predominantly based on aging individuals (>60 years old) (Lacey et al., 2019; Pichel et al., 2025; Yennurajalingam et al., 2026). In fact, the current literature on the role of exercise in chronic fatigue development during ICI therapy is quite sparse and limited to cross‐sectional studies (Pichel et al., 2025), or pilot studies with limited sample sizes (Zubac et al., 2025). Promising data were recently outlined by Yennurajalingam et al. (2026), who found that methylphenidate in combination with increased physical activity reduced cancer‐related fatigue scores in 23 patients under ICI therapy, compared to 11 controls. Therefore, a better understanding on how structured exercise mitigates chronic fatigue, especially in aging immunotherapy patients is warranted. In cancer patients receiving chemo‐ or radiotherapies, Brownstein et al. (2022), proposed that excessive fatigability may originate from exacerbated disturbances at the level of the muscle, rather than variations in voluntary activation. Testing 69 cancer patients with different diagnosis, the study utilized the—interpolation twitch coupled with EMG analysis on a custom‐made cycle‐ergometer, revealed impairments in the contractile function of the quadriceps femoris muscle. Another study by the same group found that performance fatigability, V̇O_2_ peak, TNF‐α, and age explained 35% of the variance in cancer‐related fatigue severity (Brownstein et al., 2020). Lastly, Kirkham et al. (2022) demonstrated that cancer‐related fatigue and steady‐state V̇O_2_ can vary and, at times, even show a positive correlation across a chemotherapy cycle in breast cancer patients, enabling researchers to prescribe exercise accordingly. However, it is still unknown how an individual aerobic fitness level influences chronic fatigue development in patients receiving ICI therapy compared to healthy controls. Thus, the present study investigated if chronic, cancer‐related fatigue is associated with CPET‐derived parameters (peak V̇O_2_, Peak power output, PPO) and steady‐state V̇O_2_ (e.g., submaximal cycling efforts) in immunotherapy patients of different age under acute ICI treatment compared to healthy controls. We hypothesize that the chronic fatigue would be more prevalent among immunotherapy patients and that this will partially explain their decline in CPET‐performance and cycling efficiency during submaximal, laboratory‐based aerobic exercise at gas exchange threshold (GET) compared to healthy controls.

## 2 METHODS

This study received Ethical approval from the Institutional Review Board of the Medical Faculty at the University of Cologne (reference number 13‐050). It was conducted as part of the Oncological Exercise Therapy (OTT) program, a supportive care initiative launched at University Hospital Cologne in 2012 (Sonntag et al., 2026). All procedures followed the principles of the Declaration of Helsinki. In this exploratory study, there was no a priori sample size estimation, and study participants were recruited via informational flyers outlining study details and eligibility criteria, which were distributed throughout the University Hospital Cologne, including the OTT department. Eligibility required participants to be over 18 years of age, proficient in reading and understanding German, and free from hypertension, diabetes, chronic obstructive pulmonary disease, and obesity (BMI >34.9 kg/m^2^). Patients receiving ICI therapy were recruited directly from the Center for Integrated Oncology at the University Hospital Cologne, while healthy controls of similar age were recruited from the Cologne metropolitan area. All study participants provided written informed consent and medical clearance before joining the study. For the patient group, additional inclusion criteria applied: ongoing ICI treatment as monotherapy or a combination ICI regimen (anti‐PD‐1 or PD‐L1, Keytruda©, Merck Sharp & Dohme, Opdivo©, Bristol Myers Squibb, and Imfinzi©, AstraZeneca) for a minimum of 3 months, and previous oncological treatments—such as chemotherapy, radiotherapy, or surgery—completed at least 1 month prior to participation. Exclusion criteria included the presence of metastases in the brain, bones, or lungs; concurrent immunotherapy combined with chemotherapy or radiation; a life expectancy under 3 months; and any acute orthopedic conditions or contraindications to perform moderate or vigorous aerobic exercise.

### 2.1 Data collection protocol

Data were collected in the diagnostics laboratory at the Center for Integrated Oncology, University Hospital Cologne, Germany. Participants were asked to avoid intense physical activity, caffeine, and alcohol for 24 h prior to each lab visit. Any questions or uncertainties were addressed by phone or email with the research team. A total of 52 individuals met the inclusion criteria, provided informed written consent, and visited the diagnostic lab, while 40 individuals completed all study procedures. Among those excluded for data analysis: five patients under ICI therapy completed the CPET protocol and decided to withdraw from the study; three patients were hospitalized before their second scheduled visit; two were receiving CTLA‐4‐based immunotherapy; one patient had recently undergone lung surgery and was unable to complete the CPET; while one healthy control withdrew from the study after completing the CPET. All participants included in the final analysis completed two lab visits, scheduled 48–96 h apart, following medical clearance. During the first session, anthropometric measurements were taken, and relevant medical history—particularly the immune‐related adverse events (irAEs) from the past 6 months—were documented. These data were obtained from ORBIS, the electronic health record system used at University Hospital Cologne. Next, study participants performed a ramp‐based CPET on a cycle ergometer (Ergoline 900, Hamburg, Germany), maintaining a cadence of 65–70 rpm, following previously established protocols (Zubac et al., 2025). In brief, after a 3‐min resting period, cycling began at 30 W, with the workload increasing by 15 W/min^−1^ for cancer patients and controls, until reaching exhaustion. The test ended either when participants voluntarily stopped or if their cadence dropped by more than 10 rpm for at least 10 consecutive seconds. Pulmonary gas exchange was continuously measured breath‐by‐breath using the MetaLyzer‐3B stationary cardiopulmonary testing system (Cortex, Leipzig, Germany), which was synchronized with the cycle ergometer (Ergoline, Hamburg). The metabolic system was calibrated before each session according to the manufacturer's instructions, using a standardized gas mixture and ambient air. After the test, GET was identified using the method originally described by Beaver et al. (1986) and detailed in our previous work (Zubac et al., 2022). In short, GET was determined using the MetaSoft Studio Software (Cortex, Leipzig, Germany), based on the point where a consistent rise in the ventilatory equivalent for oxygen uptake (V̇E/V̇O_2_) and end‐tidal oxygen pressure occurred, without a corresponding increase in the ventilatory equivalent for carbon dioxide (V̇E/V̇CO_2_). To account for the delay in oxygen uptake kinetics, each participant's mean response time (MRT) was used to calculate the PO associated with V̇O_2_ at GET. The oxygen uptake efficiency slope (OUES) was calculated as originally proposed by Hollenberg and Tager (2000). Specifically, the relationship between oxygen uptake (V̇O_2_, in ml/min) and total ventilation (V̇E, in L/min) was expressed using the equation: V̇O_2_ = A × log_10_(V̇_E_) + b. When V̇O_2_ is plotted on the y‐axis against the logarithm of V̇_E_ on the x‐axis, the slope of the resulting linear relationship, denoted as A, represented the OUES (Peric et al., 2025). During the second laboratory visit, participants underwent a bioelectrical impedance analysis (BIA) while lying supine after an overnight fast. They also completed the MFI‐20 questionnaire to assess chronic fatigue, followed by a 30‐min endurance test on a cycle ergometer. The test started with resting energy expenditure recording for 5 min, followed by warm up at 20 watts for 6 min, and 6 min at 90% GET performed in two rounds. Next, after 10 min of rest, a moderate‐intensity cycling was initiated, and study participants pedaled for 30 min at a constant workload matching moderate‐intensity, steady‐state cycling at 90% GET. Pulmonary ventilation and gas exchange were measured breath‐by‐breath to determine oxygen uptake (V̇O_2_), carbon dioxide production (V̇CO_2_), and the respiratory exchange ratio (RER). These values were then averaged across two‐minute intervals of each stage and used to calculate energy expenditure (kcal/min). Both gross efficiency (GE) and net efficiency of energy expenditure (EE) were calculated following standard methods (Gaesser et al., 2018); as given in Equations (1) and (2):

The gross energy expenditure and gross efficacy of moderate‐intensity cycling:

(1) $$\mathrm{GE}=\frac{\text{Power Output}}{\mathrm{EE}}\cdot 100\%$$

(2) $$\mathrm{EE}=\frac{{\mathrm{VO}}_{2}\cdot \left(\left(\frac{\mathrm{RER}-0.71}{0.29}\right)\cdot 21.4\;J\cdot {\left(\mathrm{mL}\;O_{2}\right)}^{-1}\right)+\left(\left(\frac{1.0-\mathrm{RER}}{0.29}\right)\cdot 19.4\;J\cdot {\left(\mathrm{mL}\;O_{2}\right)}^{-1}\right)\;}{60\;s}$$

### 2.2 Bio‐impedance analysis

Bioimpedance analysis (BIA) was performed using the Nutriguard‐MS by Data Input (Munich, Germany) device. All patients were tested in the morning, following a 4–5 h fast, a 12 h intense exercise fast, and a 24‐h alcohol fast. After coming to the lab, they rested supine for at least 10 min before starting the BIA measurements. The sensor electrodes were placed on the upper and lower limbs and on the opposite side of the device. Impedance was measured at 50 kHz in accordance with the manufacturer's guidelines. The collected data were analyzed using NutriPlus software (DataInpud, Munich, Germany) to calculate the lean mass/fat‐free mass of the study participants.

### 2.3 Self‐reported chronic, cancer‐related fatigue

To assess multidimensional aspects of fatigue, the Multidimensional Fatigue Inventory (MFI‐20) was used. The MFI‐20 is a validated self‐report questionnaire developed to measure fatigue across several dimensions in both clinical and non‐clinical populations, including individuals with chronic illnesses such as cancer (Schwarz et al., 2003). The questionnaire consists of 20 items, grouped into five subscales with four items each: *General Fatigue, Physical Fatigue, Mental Fatigue, Reduced Activity, and Reduced Motivation*. Participants were required to respond to each item using a five‐point Likert scale ranging from “strongly disagree” to “strongly agree.” Higher scores indicate greater levels of perceived fatigue. Each subscale yields a score ranging from 4 to 20 points. The questionnaire was administered in paper format during scheduled clinical visits and was self‐completed by the participants. Some authors use the subscale General Fatigue as a primary indicator of fatigue prevalence. We used the cut‐off scores for age‐ and sex‐standardized prevalence rates of fatigue described by Singer et al. (2011). They used MFI‐20 data from a representative German community sample (*n* = 2037) published by Schwarz et al. (2003) and calculated the age‐ and gender‐adjusted 75th percentile of General Fatigue. Patients were considered fatigued if their fatigue score was higher than that of 75% of people in the general population in the same gender and age group.

### 2.4 Statistical analysis

Raw data were stored in a Microsoft Excel spreadsheet (Microsoft Corp., 2020, Seattle, USA), and were analyzed using the JAMOVI open access tool (version 2.7.6., www.jamovi.org). Graphs were created using GraphPad Prism version 11.00 (Graph‐Pad Software, La Jolla, CA, USA). The Shapiro–Wilk test assessed the normality of data distribution. Differences between the two groups were evaluated using an independent sample *t*‐test or Mann–Whitney *U* test in case of non‐parametric data distribution. The univariate and multivariate linear regression analyses were conducted separately for controls and patients receiving ICI treatment to examine the relationships between the primary outcome variables (General fatigue item) and predictors, including the CPET‐derived parameters (relative V̇O_2_ peak and PPO) and participants' chronological age. The enter method was initially applied to all primary outcome variables to assess the predictive capacity of the full model, whereas the stepwise method was subsequently used to identify the most significant predictors of the primary outcome. Standard error of the estimate (S_EE_) was presented along with *t*‐test data, while the potential multicollinearity existence among the predictor variables was examined. Effect sizes were calculated using Cohen's d, with thresholds defined as small (*d* = 0.2), medium (*d* = 0.5), and large (*d* ≥ 0.8), and the statistical significance for all dependent variables was accepted at *p* < 0.05.

## 3 RESULTS

**FIGURE 1:**
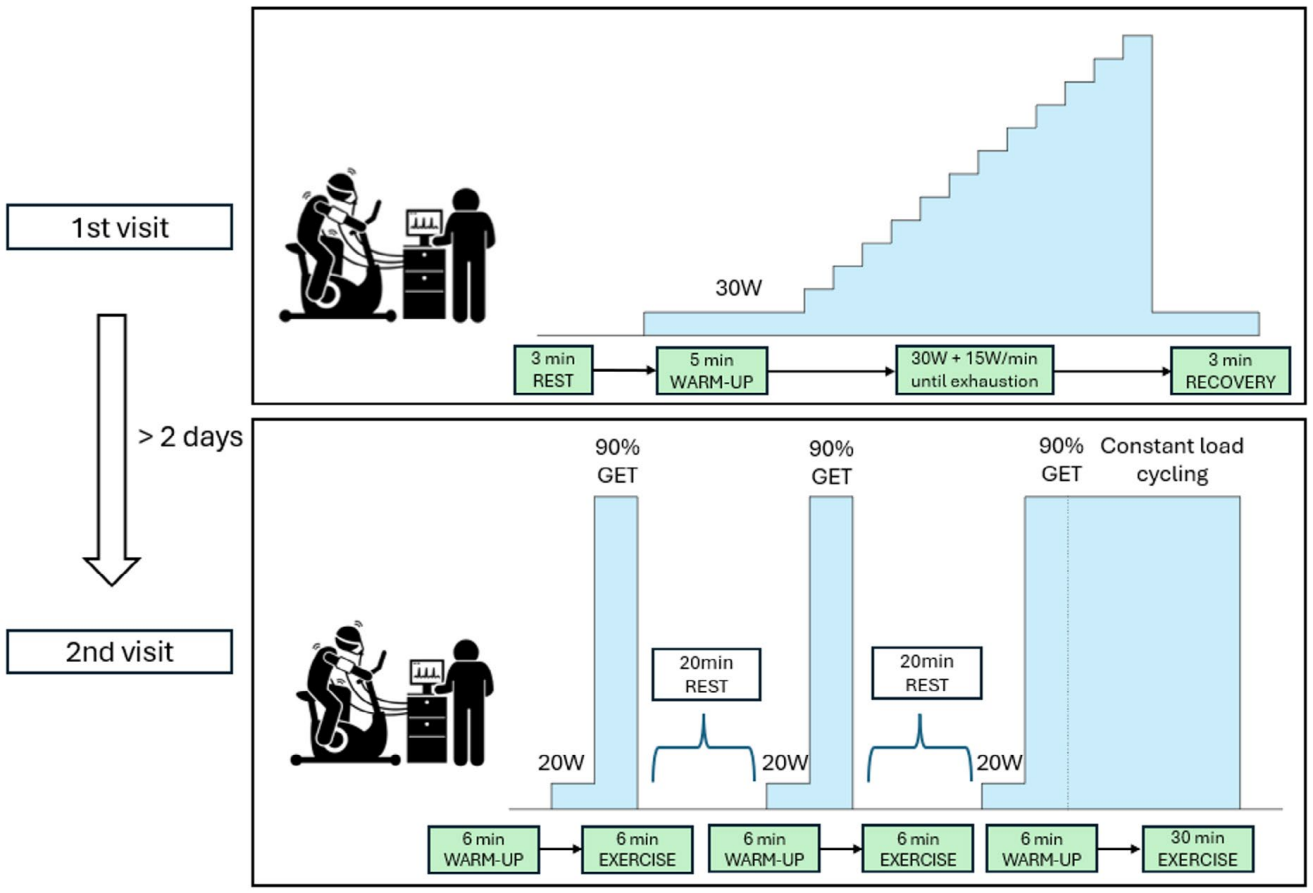
Schematic illustration of the study protocol.

**FIGURE 2:**
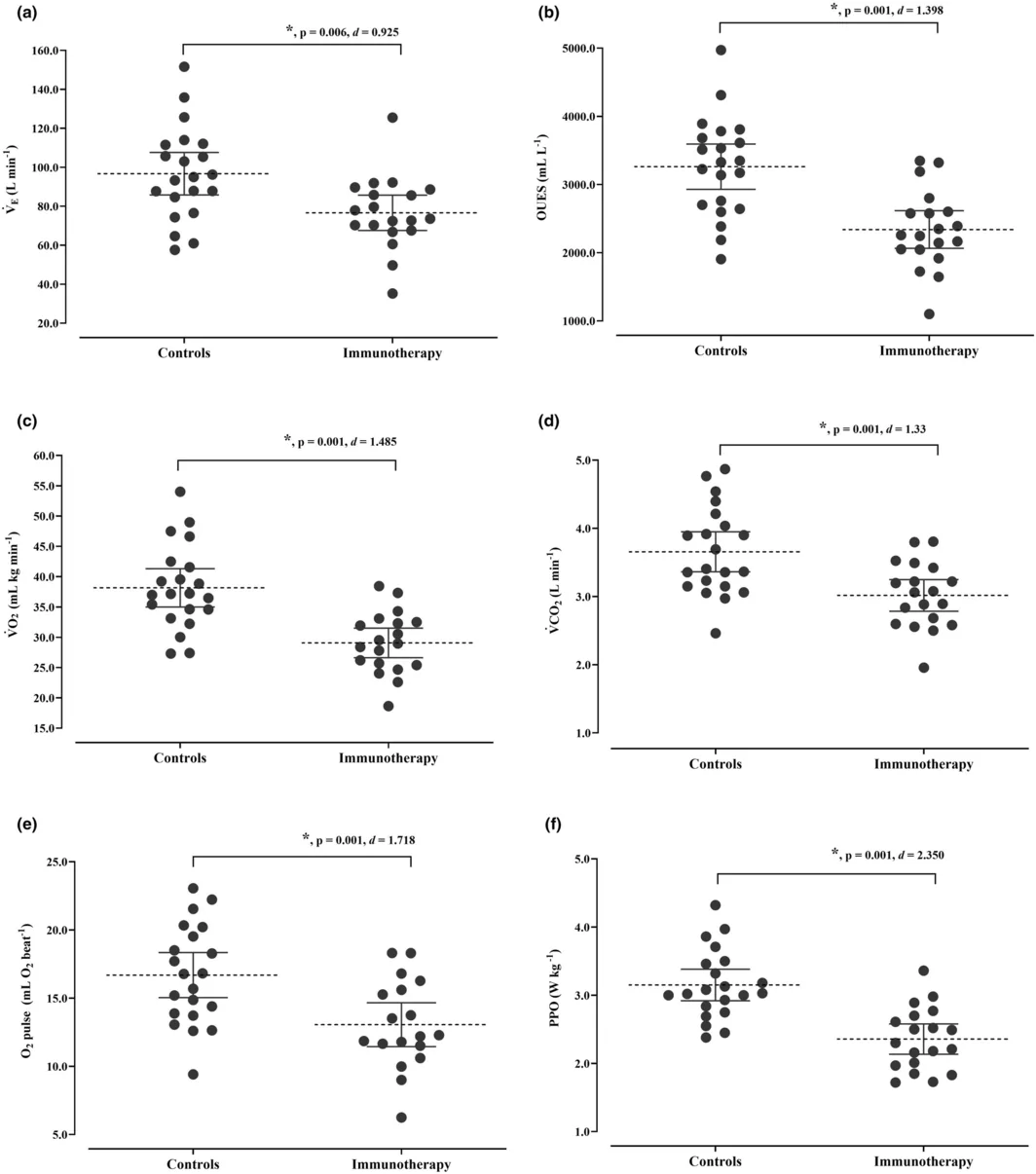
Cardiorespiratory fitness of the study participants. OUES, Oxygen uptake efficiency slope; PPO, Peak power output;V̇CO_2_, carbon dioxide production; V̇_E_, peak pulmonary ventilation; V̇O_2_ peak, peak oxygen uptake. Data are presented as mean ± SD. *Statistically different from healthy controls.

**FIGURE 3:**
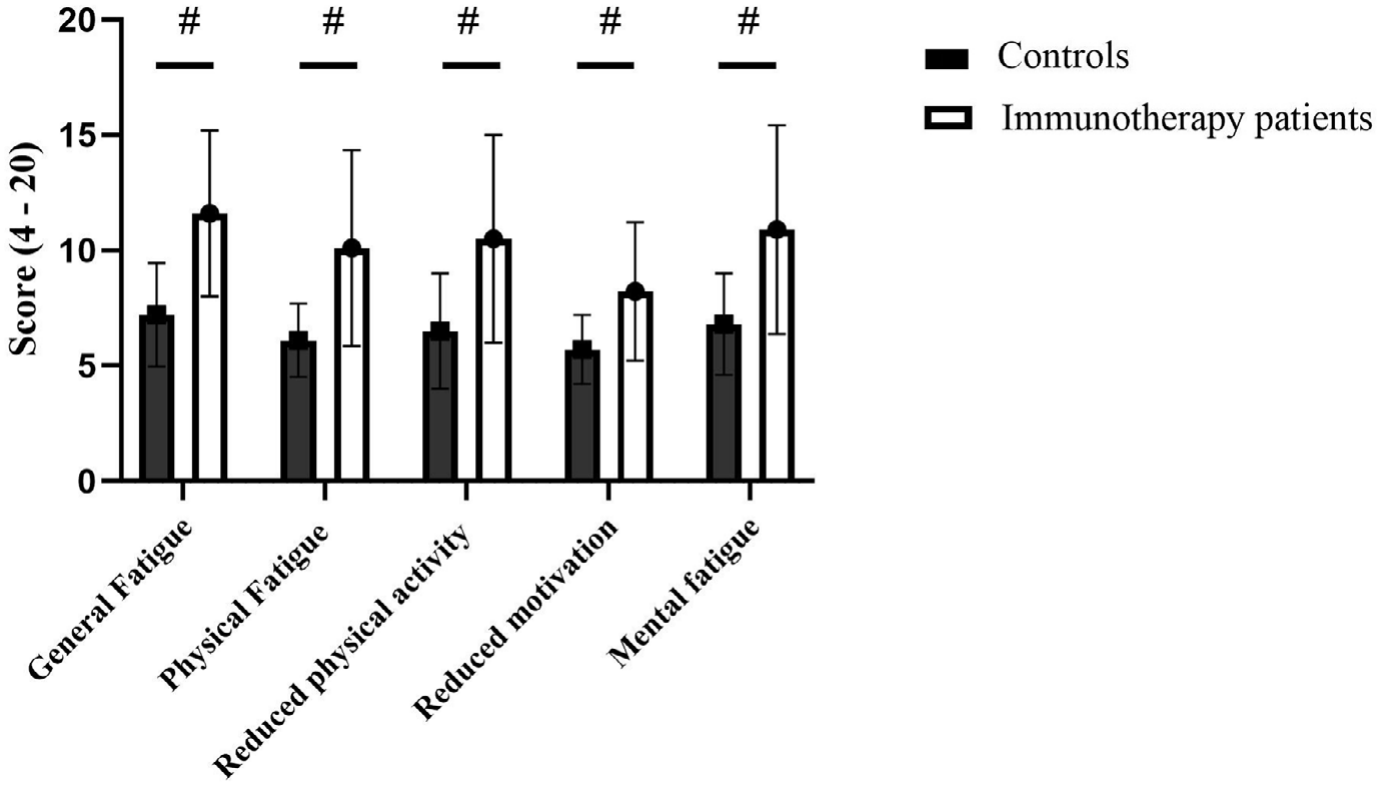
MFI‐20 chronic fatigue scores. Data are presented as mean ± SD. ^#^Statistically different from healthy controls.

**FIGURE 4:**
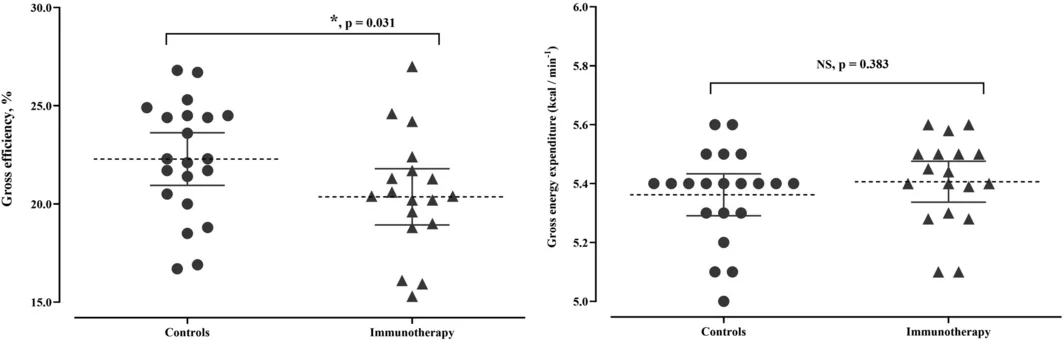
Energy expenditure and gross efficacy of the steady‐state cycling. Data are presented as mean ± SD. *Statistically different from healthy controls.

In Table 1, data on biometric characteristics of the study participants are given. Also, information regarding diagnosis and treatment of patients under acute ICI treatment are presented. There were no significant differences observed between the groups in age, stature, BMI, resting blood pressure, and oxygen saturation (*p* > 0.05, for all). Also, there were no differences observed in body composition variables derived from BIA (*p* > 0.05). The patients were receiving anti‐PD‐1 or PD‐L1 ICI therapy for 5.8 ± 2.7 months, and were on average diagnosed ~14 ± 5 months ago. Their ECOG status ranged from 0 to 1. The most common diagnoses among the studied population were malignant melanoma (21.5%) and breast cancer (21.5%), while other diagnoses included liver cancer, uveal melanoma, esophageal carcinoma, bile duct cancer, sarcoma, colon cancer, and multiple myeloma. Here, 58% of patients were graded with stage III or IV according to the TNM staging system, and 68% of patients underwent chemotherapy or radiation prior to immunotherapy treatment, while 55% had a surgery prior to ICI treatment. Only 21% of patients had distal metastasis to lymph nodes and liver. The most common ICI treatments prescribed were nivolumab (31.5%) and pembrolizumab (37%). No immune‐related adverse events (irAEs) were reported during their ICI treatment, and only a few patients reported mild colitis, classified as a non‐serious event. Tables 2 and 3 present data from linear regression models for healthy controls and patients under ICI. More precisely, in Table 2, a linear regression analysis revealed that neither peak V̇O_2_ nor age significantly predicted general fatigue scores in healthy individuals (*R* = 0.368, *R* ^2^ = 0.135, adjusted *R* ^2^ = 0.039, *p* = 0.270). The regression model explained 13.5% of the common variance in general fatigue, with a standard error of the estimate (S_EE_) of 2.21. Similarly, PPO (W·kg^−1^) and age did not significantly predict general fatigue scores in healthy individuals (*R* = 0.284, *R* ^2^ = 0.080, *p* = 0.470). The regression model explained 8.0% of the variance in general fatigue, with a S_EE_ of 2.27. In Table 3, a linear regression analysis revealed that neither peak V̇O_2_ nor age significantly predicted general fatigue scores in ICI patients (*R* = 0.237, *R* ^2^ = 0.056, *p* = 0.630). This model explained 5.6% of the variance in general fatigue, with a SEE of 3.41. Similarly, PPO (W·kg^−1^) and age did not significantly predict general fatigue scores in ICI patients (*R* = 0.206, *R* ^2^ = 0.043, *p* = 0.706), and the model explained 4.3% of the variance in general fatigue, with a S_EE_ of 3.43. All variables included in the regression models were normally distributed, and multicollinearity was not present in any of the regression models, as indicated by a variance inflation factor ranging from 1.001 to 1.15 and tolerance values ranging from 0.995 to 0.998. In Figure 1 a schematic illustration of the data collection protocol is given. In Figure 2 data on the cardiorespiratory fitness profile of the study participants are presented. The patients under ICI therapy had lower readings of V̇_E_, V̇O_2_, V̇CO_2_, stroke index, and OUES (all *p* < 0.001, with large effect sizes, *d* = 0.92–1.33). Also, their CPET was ~4 min shorter compared to controls, and PPO reached was significantly lower (*p* = 0.001, *d* = 2.35), while no difference was observed in their self‐reported RPE (on average 19 ± 1). In Figure 3, differences in self‐reported fatigue items are given. Among patients receiving ICI therapy, 63% of study participants were classified as chronically fatigued, compared with 15% of the healthy population who self‐reported chronic fatigue. Distribution normality was violated in all variables except for the general fatigue item. The immunotherapy group self‐reported significantly higher levels of general fatigue (by 38.1%, *d* = 1.48), physical fatigue (by 39.7%, rank biserial correlation, rrb = −0.556), reduced physical activity levels (37.9%, rrb = −0.511), reduced motivation (by 30%, rrb = −0.508) and mental fatigue 38.2% (rrb = −0.537). Lastly, in Figure 4, the independent group *t*‐test revealed no statistically significant differences between the groups for the energy expenditure, *t*_(38)_ = −0.930, *p* = 0.358, representing a small effect size magnitude (*d* = −0.294). In contrast, a statistically significant group difference was observed for the GE% parameter, *t*_(38)_ = 2.246, *p* = 0.031. The mean difference between the groups was 2.16, while the effect size (*d* = 0.711) was moderate to large. Interestingly, all study participants completed the 30‐min steady‐state cycling at 90% gas exchange threshold, with average PO of 122 W for controls, and 88 W for patients under ICI treatment.

**TABLE 1:** Biometric characteristics of the study participants.

	Controls *n* = 21 (43% ♀)	Immunotherapy *n* = 19 (52% ♀)	*t*‐test	Mean difference	*p*‐value	*d*
Age, years	47.7 ± 17.1	47.9 ± 14.3	−0.035	−0.180	0.972	−0.001
Body height, cm	174 ± 7	175 ± 9	0.600	1.519	0.552	0.190
Body mass, kg	74.7 ± 12.2	72.7 ± 14.3	0.456	1.982	0.650	0.147
BMI, kg·m^−2^	24.4 ± 2.6	24.1 ± 4.1	0.234	0.262	0.814	0.075
Resting SBP, mmHg	117 ± 14	109 ± 14	1.806	8.000	0.079	0.572
Resting DBP, mmHg	77 ± 9	73 ± 9	1.215	3.625	0.323	0.385
Resting MAP, mmHg	90 ± 10	85 ± 9	1.799	5.486	0.080	0.569
SpO_2_, %	98 ± 1	98 ± 1	0.605	0.135	0.548	0.198
Fat free mass, kg	59.6 ± 11.1	54.3 ± 10.9	1.551	5.32	0.129	0.485
Fat mass, %	21.6 ± 6.01	24.5 ± 9.8	−1.131	−2.83	0.267	−0.325
Diagnosis related parameters						
Time since diagnosis (months)	—	14.5 ± 5.2				
Therapy duration (months)	—	5.8 ± 2.7				
Cancer stage (%)						
I	—	5	—			
II	—	6	—			
III	—	4	—			
IV	—	4	—			
Immunotherapy regime						
Nivolumab (PD‐1)	—	6	—			
Nivolumab + Ipilimumab	—	2	—			
Pembrolizumab (PD‐1)	—	7	—			
Durvalumab (PD‐L1)	—	3	—			
Durvalumab (PD‐L1) + TT (Daratumab CD38)	—	1	—			

*Note*: Data are presented as mean ± SD.

Abbreviations: BMI, body mass index; *d*, effect size; DBP, diastolic blood pressure; MAP, mean arterial pressure; SpO_2_, oxygen saturation; SBP, systolic blood pressure.

**TABLE 2:** Linear regression analysis of healthy individuals.

Predictors	*B*	SE *B*	*β*	95% CI	*t*‐test	*p* ≤ 0.50
General fatigue (4–20)						
Constant	4.072	3.46	—		1.177	0.255
Age, years	−0.017	0.225	−0.127	−0.076 to 0.042	−0.564	0.580
V̇O_2 peak_ (mL·kg^−1^·min^−1^)	0.102	0.226	0.316	−0.041 to 0.245	1.399	0.179
General fatigue (4–20)						
Constant	5.028	4.211	—		1.194	0.248
Age, years	−0.017	0.242	−0.127	−0.080 to 0.046	−0.525	0.606
PPO (W·kg^−1^)	0.939	0.243	0.212	−1.170 to 3.048	0.873	0.394

Abbreviations: *β*, standardized beta coefficient; 95% CI, confidence intervals; *B*, unstandardized beta coefficient; V̇O_2_ peak, peak oxygen uptake.

**TABLE 3:** Linear regression analysis of immunotherapy patients.

Predictors	*B*	SE *B*	*β*	95% CI	*t*‐value	*p* ≤ 0.50
General fatigue						
Constant	12.982	5.62	—		2.309	0.035
Age, years	−0.206	0.058	−0.206	−0.695 – 0.283	−0.826	0.420
V̇O_2_, (mL·kg^−1^·min^−1^)	0.030	0.159	0.030	−0.440 – 0.500	0.125	0.901
General fatigue (4–20)						
Constant	15.891	5.51	—		2.88	0.010
Age, years	−0.053	0.246	−0.225	−0.167 – 0.061	−0.912	0.374
PPO (W·kg^−1^)	−0.876	0.246	−0.121	−4.352 – 2.600	−0.494	0.627

Abbreviations: *β*, standardized beta coefficient; 95% CI, confidence intervals; *B*, unstandardized beta coefficient; PPO, peak power output; V̇O_2_ peak, peak oxygen uptake.

## 4 DISCUSSION

The primary aim of the present study was to investigate if cancer‐related fatigue is associated with CPET‐derived parameters in patients under ICI treatment and healthy controls. Next, the secondary aim was to examine if cancer fatigue prevalence in the group under ICI treatment would affect their energy expenditure and cycling efficiency during submaximal, 30‐min efforts. Our findings can be summarized as follows: the patients under ICI self‐reported higher levels of chronic, cancer‐related fatigue, lower cardiorespiratory fitness level, and reduced cycling efficiency compared to healthy controls. This is the first study to explore the link between chronic, self‐reported cancer fatigue and aerobic capacity among ICI patients. Data presented here have confirmed what we already know regarding patients under ICI during acute treatment, to some extent. These patients are characterized by higher levels of general and physical fatigue (Figure 3, (Yennurajalingam et al., 2026)), and exhibit lower levels of self‐reported physical activity during acute immunotherapy treatment (Pichel et al., 2025). The self‐reported MFI‐20 readings reported here were similar to those reported in cancer patients of similar age under immunotherapy (Yennurajalingam et al., 2026) or even acute chemotherapy (Demmelmaier et al., 2021). In parallel, their cardiorespiratory fitness levels were significantly lower compared to healthy controls (Figure 2). The cardiorespiratory fitness profile was characterized by reduced V̇O_2_ peak, lower O_2_ pulse, and lower OUES in the ICI group compared with healthy controls. These findings can be interpreted as an overall indicator of impaired central O_2_ delivery capacity. Apparently, a reduction in O_2_ pulse observed during CPET, together with only modest differences in maximal heart rate suggests a limitation in stroke volume augmentation and most likely reduced cardiac output during maximal efforts. Peak PO and time to exhaustion during CPET were significantly lower in the patient group compared with healthy controls. Data presented here are in line with other cancer patient populations under acute treatment (Foulkes et al., 2024), thereby highlighting exercise intolerance, and may suggest cardiotoxicity development in this population, as a consequence of their previous treatment, in combination with current ICI therapy. This hypothesis is quite plausible, and present findings (Figure 2) suggest a predominantly central cardiovascular limitation to exercise performance, contributing to the observed reduction in functional capacity observed during CPET. However, since there were no specific diagnostic procedures applied to track cardiotoxicity development in this exploratory study, our suggestion remains theoretical and requires further research.

The regression analysis (Tables 2 and 3) found no association between the self‐reported MFI‐20 general fatigue item and objectively measured CPET parameters– V̇O_2_ and PPO. Indeed, the regression models reported here explained between 5.5% and 13% of the common variance for dependent variable(s) (*p* > 0.05) in both groups, leaving an open question regarding relevant predictors that could predict the self‐reported fatigue development. For example, age was not a significant predictor, despite the fact that aging is often associated with a decline in aerobic fitness and overall physical performance, and most of the work published in exercise and immunotherapy is based on a population of older individuals. In similar work, using a longitudinal design, Mallard et al. (2025) outlined a regression model explaining 39% of the common variance among the FACIT‐*F* score – a measure of self‐reported fatigue, and predictors consisting of 6‐min walking test distance, knee extensor muscle force, neuromuscular fatigue, and physical activity level in female breast cancer patients under acute chemotherapy. Important differences between our work and the work of Mallard et al. (2025) include differences in study design (cross‐sectional vs. longitudinal), type of data collected (objective measures of aerobic performance outlined here and lack of neuromuscular data in our work) and lastly, the number of patients included. Still, this cannot entirely explain the difference between the studies, as we recently observed no change in MFI‐20 derived fatigue scores following exercise interventions in a crossover study design where we collected CPET data in male breast cancer patients (Schultz et al., 2023). Thus, the data presented here on the underlying mechanisms of chronic, cancer‐related fatigue development appear to be quite complex (Twomey et al., 2017) in general, and especially under ICI treatment. The unclear pathogenesis and additional, implicated drivers of cancer‐related fatigue such as circulating inflammatory cytokines, changes in muscle metabolism, and neurotransmitter disruption illustrate the difficulty in fatigue research and management (Bower, 2014; Peters et al., 2021). In addition, chronic fatigue pathogenesis seems not to be only multidimensional, but also interindividual different with multiple and different fatigue subtypes as reported by Schmidt et al. (2024). Therefore, future studies could integrate fatigue subtype classifications/assessments to gain further, more detailed insight of physical performance in chronic fatigue pathogenesis in cancer patients receiving ICI treatment.

A quite novel and unique finding of our study was the reduced cycling efficiency in ICI patients compared to healthy controls. We found significant differences in GE% between the two groups (Figure 4, panel A), indicating that cycling efficiency is impaired during submaximal cycling efforts in patients under ICI. Interestingly, this was not translated into greater task‐failure at 90% GET, as all study participants completed the 30 min cycling task. Previous work in this line of research focused on the effects of age, sex, and training status in healthy individuals, concluding that cycling efficiency does not decline due to differences in age, sex, or training status (Duong et al., 2024). This is further confirmed by data recently reported by Zoladz et al. (2025), who reported GE of ~22% in young healthy untrained men cycling at 120 W, that aligns with data reported for healthy individuals. Since there are no similar data in patients under ICI, and we cannot make a direct comparison with such population, we can only assume that the lower cycling efficiency in patients under ICI is due to impaired mitochondrial‐coupling efficiency as others have observed in using in vivo _31_P magnetic resonance spectroscopy (Conley et al., 2013). Moreover, recent biopsy findings of vastus lateralis from Mallard et al. (2024) support our hypothesis to some extent, as the authors found that a single dose of chemotherapy administration in breast cancer patients is translated into mitochondrial dysfunction. Since data on the mitochondrial dysfunction of patients under ICI treatment are not yet available in the literature, additional research in this area is warranted. This dataset provides a basis for designing and recommending aerobic exercise interventions for patients undergoing acute ICI treatment. In the present sample, 63% of patients receiving ICI therapy were characterized by excessive fatigue, yet they were still able to complete a 30‐min cycling task at 90% of GET. This suggests that, despite clinically relevant fatigue, moderate‐to‐vigorous aerobic exercise may remain feasible and tolerable in this population under controlled conditions. Furthermore, previous studies in immunotherapy‐treated patients with different diagnoses (Yennurajalingam et al., 2026), have shown that structured exercise interventions can reduce chronic self‐reported fatigue by approximately 8–10 points on validated fatigue scales. Taken together, these findings support the role of individualized aerobic exercise prescriptions as a complementary strategy to mitigate chronic fatigue during ICI therapy, although confirmation in a larger number of patients is warranted. This is especially important since structured exercise interventions are recommended as first‐line therapy to mitigate chronic, cancer‐related fatigue (Mustian et al., 2017).

### 4.1 Study limitations

Some limitations to our study should be mentioned. Firstly, a cross‐sectional design and relatively small sample size limit the generalizability of the findings and increase the risk of type II error. Furthermore, the heterogeneity of cancer diagnoses could be seen as a potential confounding factor, while the lack of direct cardiac and mitochondrial measurements should be considered when interpreting the results. The heterogeneity of previous anticancer treatments limits the ability to distinguish the specific effects of immune checkpoint inhibitor therapy from potential treatment‐related effects. Accordingly, the findings should be interpreted as characterizing patients receiving immunotherapy checkpoint inhibitors (ICI) treatment rather than demonstrating causal effects of immunotherapy. Future studies with a larger sample size, objectively measured physical activity levels, and comprehensive mechanistic assessments are therefore warranted.

## 5 CONCLUSION

This was a first study to explore the link between self‐reported, cancer‐related chronic fatigue and aerobic fitness in patients under acute ICI therapy. In general, the patient population reported higher levels of chronic fatigue, lower V̇O_2 peak_, and reduced cycling efficiency compared to healthy controls. The parameters collected during CPET did not explain self‐reported chronic fatigue, underlying the complexity of this phenomenon. Still, this study goes one step ahead of the previously published feasibility and adherence studies by providing first data on work efficacy of patients under ICI treatment and depicting recommendations for aerobic exercise for this patient population. Future research should prioritize exercise testing using more comprehensive, non‐invasive techniques like the flow mediated dilation or vascular occlusion test to provide more detailed information on the oxygen transport pathway cascade in this specific group of patients.

## AUTHOR CONTRIBUTIONS

**Damir Zubac:** Conceptualization; data curation; formal analysis; funding acquisition; investigation; methodology; resources; software; supervision; validation; visualization. **Timo Sonntag:** Conceptualization; data curation; formal analysis; methodology; project administration. **Diana Kranjc:** Conceptualization; formal analysis; project administration; resources; software; validation; visualization. **Freerk T. Baumann:** Conceptualization; project administration; resources; supervision; validation; visualization.

## FUNDING INFORMATION

The article processing charge was covered from the project (Chronic Fatigue Management in Immunotherapy: A German–Slovenian Collaboration for Precision Exercise Oncology) funded by Cologne International forum, University of Cologne., PI Dr. Zubac, D.

## CONFLICT OF INTEREST STATEMENT

The authors declare no competing interests.

## CONSENT

All study participants provided written consent.

## DISCLOSURE

The authors confirm that generative artificial intelligence tools were not used in the preparation of this manuscript.

## ETHICS STATEMENT

This study received Ethical approval from the Institutional Review Board of the Medical Faculty at the University of Cologne, reference number 13‐050.

## Data Availability

Data collected and analyzed in this study are available from the corresponding author upon reasonable request.
